# Research on Voice-Driven Facial Expression Film and Television Animation Based on Compromised Node Detection in Wireless Sensor Networks

**DOI:** 10.1155/2022/8563818

**Published:** 2022-01-24

**Authors:** Shi-Jiang Wen, Hao Wu, Jong-Hoon Yang

**Affiliations:** ^1^Department of Digital Image in Sangmyung University, Seoul 03015, Republic of Korea; ^2^Department of Xinjiang Art and Sports, Ningbo Childhood Education College, Ningbo 315016, China; ^3^School of Animation and Digital Arts, Communication University of Zhejiang, Hangzhou 310018, China

## Abstract

With the continuous development of social economy, film and television animation, as the spiritual needs of ordinary people, is more and more popular. Especially for the development of emerging technologies, the corresponding voice can be used to change AI expression. But at the same time, how to ensure the synchronization of language sound and facial expression is one of the difficulties in animation transformation. Relying on the compromised node detection of wireless sensor networks, this paper combs the synchronous traffic flow between the speech signals and facial expressions, finds the pattern distribution of facial motion based on unsupervised classification, realizes training and learning through neural networks, and realizes one-to-one mapping to facial expressions by using the rhyme distribution of speech features. It avoids the defect of robustness of speech recognition, improves the learning ability of speech recognition, and realizes the driving analysis of facial expression film and television animation. The simulation results show that the compromised node detection in wireless sensor networks is effective and can support the analysis and research of speech-driven facial expression film and television animation.

## 1. Introduction

With the continuous development of social economy, film and television animation is more and more sought after and favored by many people, especially rural people. It is not only the consumption of spiritual culture but also an embodiment of national culture [1, 2]. The development of artificial intelligence and other technologies has promoted the development of language pronunciation and facial expression, especially to meet different language pronunciation and interaction with different people; that is, language pronunciation is one person, and facial expression is another person [3, 4]. There is a close synchronous relationship between the two, but how to realize the seamless connection between the two and realize that language pronunciation directly drives facial expression is the research focus and difficulty of human-computer interaction under various modes, and many industry experts have conducted corresponding research [5, 6]. Generally speaking, the driving of facial expression by speech can be mainly through speech pronunciation recognition and other types of recognition. For speech pronunciation recognition, the corresponding eigenvalues are mainly used for analysis, especially for the basic feature units of speech recognition based on factors and syllables. Mature empirical rules drive a whole set and use corresponding eigenvalues to compare corresponding lip shapes. This method is relatively simple and effective, but it is limited by rules. This rule requires manual definition by various experts and is limited to experts' experience and knowledge. Therefore, qualitative analysis is not accurate enough [7, 8]. At the same time, it is very difficult to realize accurate nonqualitative human speech recognition. This requires directly extracting the corresponding speech features and directly mapping them to the specific parameters of the face. Therefore, this method can not only avoid the problems encountered in speech pronunciation to a certain extent but also effectively realize synchronization and realism [9, 10].

Some scholars proposed to use artificial intelligence and corresponding prediction to realize the training and learning from speech to facial expression and realize the tracking and relative position of facial expression by inputting relevant parameters [11, 12]. In addition, some scholars have proposed to use the relevant methods of neural networks to synthesize vision and speech, realize the recognition and analysis of speech and face motion parameters, and obtain a better feedback effect [13, 14]. Other scholars used the Markov chain for control analysis and introduced speech recognition for driving analysis of facial animation, that is, getting facial expression motion according to corresponding computer vision analysis technology and realizing learning and control facial expression model. This method can effectively solve visual synchronization on the one hand and human motion behavior on the other hand and realize the expression animation of face driven by voice pronunciation, but in the actual application process, there are still many limitations and specificities [15, 16].

In view of these limitations and requirements, this paper introduces wireless sensor network compromised node detection, combs the synchronous service analysis of voice pronunciation and facial expression, uses unsupervised classification technology to accurately analyze the corresponding facial expression, and realizes manual lip shape adjustment through voice level clustering analysis. At the same time, the video motion data can be used to train the voice to drive the face animation model, improve the authenticity and fidelity of the system, reduce the defects of artificial rules, and use the corresponding rhythm to directly add and map to the face pattern to realize the final driving analysis of face expression film and television animation.

## 2. Compromised Node Detection Method for Wireless Sensor Networks

By comparing the disadvantages and advantages of speech recognition and nonspeech recognition, this paper selects the latter so that neither the discreteness of speech samples nor the parameter mode of speech directly mapped to face motion needs to be considered [17, 18].

Detection using corresponding wireless sensor nodes can be mainly divided into the following steps:

1. In the whole network, it is necessary to focus on deploying corresponding sensor nodes to ensure that sensor nodes have overlapping coverage. Therefore, it can be predicted that the occurrence or generation of an element may be detected by multiple nodes, and the node of one sensor can also detect multiple behaviors of other adjacent nodes [19, 20].
2. For ordinary and distributed sensor nodes, they can only have limited ability and communication ability.
3. For the transmission channel between the node and the base station, it is necessary to abide by the corresponding transmission routing protocol.
4. For the base station, it is a computing device capable of representing location information.
5. For the sensor node, it needs to have enough identifiers to represent it. In the whole deployed network, it needs to compromise according to the corresponding nodes.
6. All messages are time stamped.
7. The attacker needs to capture the physical address to obtain the corresponding site location and information.

For the base station site, if there are more resources, it is more secure and difficult to overcome. However, it should be noted that the network may transmit false data.

**Figure 1:**
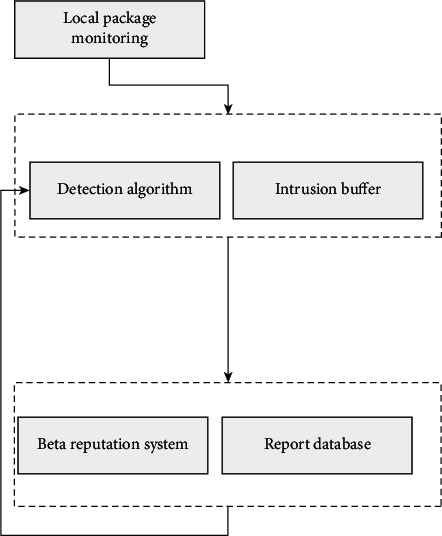
Frame structure.

The whole system can be composed of two parts: one is a distributed network site system, and the other is a centralized system running in the so-called related base stations (Figure 1).

The so-called distributed component is relative to the deployment environment of the network. Each copy of its component runs on different network sensor nodes and is synchronized with the relevant transmission protocol and transmission app on the sensor. When each node detects the possible compromised behavior in adjacent nodes, it needs to report to the nearest base station for transmission in time. This detection method needs to rely on adjacent nodes for detection, and each node needs to detect and analyze adjacent nodes. However, due to the characteristics of wireless sensor networks, such deployment saves a certain cost.

For centralized components, they are higher-level computing devices, so it may be necessary to perform more complex behavior analysis to summarize whether there are corresponding compromised behaviors to the center and whether these compromised behaviors are legal. For the corresponding data collected by the whole network, these are collected and analyzed through their own network resources.

### 2.1. Distributed Component

Each sensor node has a distributed component running on it, which will record the data from adjacent nodes and establish a baseline based on these records. If a neighbor node continues to perform abnormal behavior, it will be identified as a compromised node and reported to the base station.

**Figure 2:**
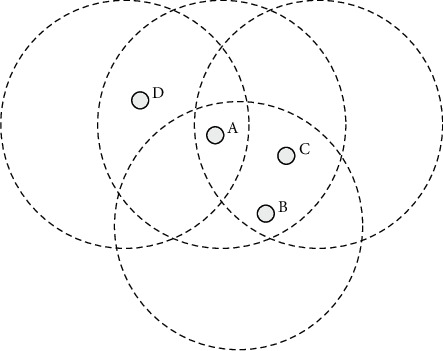
If node *a* wants to impersonate any node, it must pass the detection of neighbor nodes.

As shown in Figure 2, if the corresponding node A is attacked and considered by the attacker to imitate another node and if the neighbor node D does not detect A, then node A cannot directly imitate B or C. In this way, the node has enough neighbors to be solid with each other so that any attack and impersonation can be detected in time.

Considering instantaneous errors, such as collisions or other nonmalicious behaviors, ComDet has certain flexibility when determining a node as a compromised node and can tolerate certain abnormal behaviors. When judging whether a neighbor node behaves abnormally, there is no cooperation between nodes. This independent decision-making process means that the compromised node cannot affect the perspective of the legitimate neighbor node.

#### 2.1.1. Monitoring Indicators

The first step in designing any detection-based security system is to select the characteristics of the system that will be monitored. In order to support most wireless sensor networks, ComDet only monitors some common features of wireless sensor networks.

  ① Sensor readings: by monitoring sensor readings, attacks that attempt to distort the collected information can be detected.
  ② Received power: in a static network, the received power should remain unchanged. Fluctuations may be caused by changes in communication hardware or the location of corresponding nodes.
  ③ Sending rate: most applications read sensor readings and send them periodically. Any routing data packets will also be sent periodically. Therefore, the rate of data packets sent by a node should follow a consistent pattern. Most attacks, such as selective forwarding, Sybil attacks, and replay attacks, will cause measurement bias. In addition, the sudden idle period may be caused by the opponent's reprogramming of the node.
  ④ Receive rate: the ratio of incoming and outgoing data packets should be constant because the outgoing data packets can only be data packets routed by the node or generated by the node. A neighbor node that changes its receiving rate but does not change its sending rate may be a compromised node. It should be noted that regardless of whether the data is encrypted or not, the header of the data packet can usually be seen by all nodes.

Because most wireless sensor networks have these characteristics, ComDet has a wide range of applicability. However, these features may not be appropriate in two situations: (1) data packets can only be decrypted by the base station; (2) applications rarely exchange information with the base station.

When the confidentiality of information transmitted on the network is very important, the first scenario will appear. Since the compromised node cannot be detected and blocked immediately, some of the information sent may be eavesdropped on by the compromised node. Therefore, the data packet can be encrypted, and only the base station can decrypt it. Under such conditions, the defect of not being able to monitor sensor readings can be compensated by increasing the number of monitored neighbors so that ComDet can achieve corresponding performance by occupying more memory.

The second scenario is caused by the application of nonperiodic communication. For example, wireless sensor networks located in the quarantine zone only send information when an attack is detected, and they will not communicate in a secure environment. Because the monitored information is not sufficient, it is impossible to establish a baseline for most features. ComDet compensates by making the node send its unique identification code at a certain speed. In order to avoid prolonged silence and fail to discover that the node has been conquered by the attacker, a certain amount of communication overhead is necessary. This mode of behavior is only used when the application has very little communication volume.

#### 2.1.2. Detection Algorithm

There are two types of algorithms for detecting abnormal behavior: anomaly detection and rule-based detection. They all use records of monitoring system characteristics. In anomaly detection algorithms, the existing records are first used to establish a baseline, and any new records that deviate from the baseline to a certain extent are considered abnormal behaviors. On the contrary, rule-based detection requires the establishment of a specific standard. For example, if any two packets have the same header, it means that a replay attack has occurred. In the ComDet system, the main focus is on anomaly detection algorithms to meet ComDet's requirements for flexibility, rule-based algorithms target specific situations, and the rules they use must be updated continuously for each new situation.

ComDet's distributed components can be divided into five algorithms for attack detection: the first four are anomaly detection algorithms, which use network characteristics such as sensor readings, receiving power, sending rate, and receiving rate; the fifth is a rule-based detection algorithm.

For rule-based algorithms, if a node detects that a new neighbor meets the characteristics in the preset rule base based on the received message, it will consider the node to be a compromised node.

For anomaly detection, we need to identify the abnormal behavior. Based on the abnormal behavior of each node, we need to set different buffers, one is the buffer for normal packets, and the other is the buffer for abnormal behavior. When all detected abnormal phenomena are shared in the exception storage, the window is used for sliding analysis and compared with the corresponding limit. If it exceeds a certain security threshold, the data packet is considered abnormal. Abnormal behavior may have certain intrusion behavior, which will also cause the behavior report of the node.

**Figure 3:**
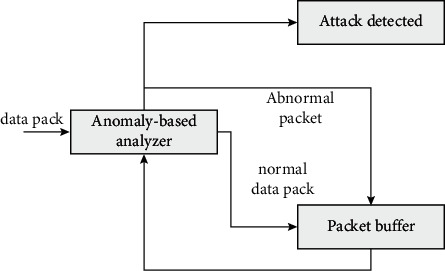
Overview of detection algorithms using received power and sensor readings.

The specific overview of the received power is shown in Figure 3, and the specific calculation equation is shown in the following formula:

(1) $$\begin{array}{c} {\text{Power}}_{\text{new}}-{\text{power}}_{\text{max}}>T,\text{if }{\text{power}}_{\text{max}}>{\text{power}}_{\max},\\ {\text{power}}_{\min}-{\text{Power}}_{\text{new}}>T,\text{if }{\text{power}}_{\text{new}}>{\text{power}}_{\min}. \end{array}$$

Compromised node detection in wireless sensor networks is used to analyze the maximum and minimum interval values of the received power of the packet buffer. When the received power of a new packet exceeds this interval, it is considered that the behavior is abnormal. Therefore, the abnormal data needs to be divided into abnormal packet buffer. After processing, the corresponding abnormal situation processing caused by environmental change is realized.

**Figure 4:**
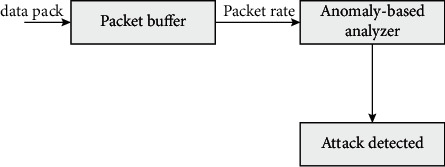
Overview of detection algorithm using transmission rate and reception rate.

As shown in Figure 4, it is a conceptual diagram of using transmission power, which is mainly used to calculate the transmission rate of data packets and the transmission rate of data packets. When the ratio of the two rates exceeds the set threshold range, the adjacent neighbor nodes can be considered as compromised nodes.

The algorithm using the reception rate is different only in two aspects.

All the illegal behaviors of a node detected by the anomaly detection algorithm are stored in a shared illegal behavior buffer.

Once the abnormal behavior of a neighbor is detected, the weight of its abnormal behavior is calculated as shown in the following formula:

(2) $$\begin{array}{c} \sum_{M}\left(t_{\text{current}}-t_{\text{stamp}}\right)+0.3\sum_{m}\left(t_{\text{current}}-t_{\text{stamp}}\right). \end{array}$$

Once a node determines that a neighbor node *a* is compromised, it will send three reports about the compromised node to the base pair.

### 2.2. Centralized Component

The centralized component runs on the base station and judges whether a reported node is really compromised based on the data from other nodes.

*f*(*ρ* *|* *α*, *β*) can be calculated with Γ function, and its calculation formula is shown in the following formula:

(3) $$\begin{array}{c} f\left(\rho \;|\;\alpha ,\beta \right)=\frac{\Gamma \left(\alpha +\beta \right)}{\Gamma \left(\alpha \right)+\Gamma \left(\beta \right)}\rho^{\alpha -1}{\left(1-\rho \right)}^{\beta -1},\;0\leq \rho \leq 1,\;\alpha >0,\beta >0. \end{array}$$

The facial motion parameter FAP (facial animation parameter) defined by MPEG-4 is a set of parameters for realizing facial animation. FAP is based on the subtle movements of the face, and through detailed description and quantification of the actions of various parts of the face model, it can reproduce most natural facial expressions and lip movements. The MPEG-4 standard defines a total of 68 FAPs, including lip shape (viseme) FAP and expression (expression) FAP. For these two FAPs, some basic and different phantoms or expression data are stored in advance, and other phantoms or expressions can be formed by a linear combination of these basic phantoms or expressions. The function of lip shape and expression FAP is to accurately and conveniently express simple lip movements and expressions, but for complex and irregular lip movements and expressions, lip shape and expression FAP are difficult to describe well. Therefore, in the MPEG-4 standard, in addition to lip shape and expression FAP, it is more convenient for users to describe. For subtle facial movements, general FAP definitions are also given. Generally, FAP is mainly used for movements in a specific area of the face, such as raising eyebrows and moving the upper lip up. The general FAP includes details of each part of the face motion description, which can generate more complete and complex animations than the 14 lip shapes and expression FAP defined by the standard. Therefore, the FAP mode in this paper is based on the general FAP. At the same time, it is different from the basic lip shapes and expressions defined by MPEG4. The FAP model described in this paper does not refer to hypothetical speech perception classification, such as cluster analysis of phonemes or words, but is directly obtained by cluster analysis from a large amount of real image data, so that the results obtained can be used more effectively. In face animation, we assume that all possible face poses belong to a certain state in a high-dimensional space plane, and complex face motion can be regarded as a transition between thousands of states.

Aiming at this diverse and complex face motion trajectory, our learning strategy is to use some FA patterns that can effectively characterize the face motion characteristics or represent a certain set of states to approximate this diversification in segments and then use linear interpolation to glue this segmentation to achieve high-fidelity facial animation. Of course, to achieve voice-driven facial animation, the acquisition of these FAP mode sequences must be obtained through a certain prediction method. At the same time, due to the complexity of face motion, only through the results learned from the large-scale audio and video library can we realize more natural face animation. Manual rules cannot meet the requirements. Because neural networks have better characteristics in processing input and output mapping relations and meet the characteristics of the human brain structure, we use neural networks to learn this mapping relationship.

### 2.3. System Framework

**Figure 5:**
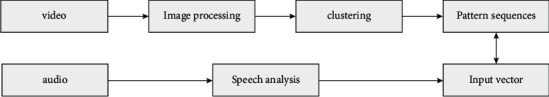
Speech-driven facial animation synchronization processing framework.

As shown in Figure 5, it is a specific step of synchronous processing of voice-driven facial animation. Using the corresponding technologies such as computer vision tracking, image processing, and wireless sensor network compromised node detection, the features of corresponding facial parameters and facial animation parameters are extracted from the video and calculated according to the corresponding eigenvalues, and the corresponding patterns are obtained by clustering method. By analyzing the learning context, the mapping relationship of learning is obtained, and the effective analysis and synchronization of speech and face animation are realized.

## 3. Data Preprocessing

### 3.1. Speech Signal Analysis

There are many kinds of speech signal processing. How to extract the feature elements in speech recognition is not completely effective for the prediction of facial expression, especially how to achieve synchronization, which is worth thinking and analyzing. Speech pronunciation and face behavior recognition may be related to speech and corresponding feature elements, which can ensure complexity and looseness. In the data preprocessing of this paper, different speech features are used for analysis and processing, and the association of prosody and facial expression in speech is used to obtain the pitch stream and ability analysis in speech segments, which are finally synthesized to form a unique feature vector.

### 3.2. Video Signal Analysis

In order to uniformly obtain the feature data of facial expression, this paper synchronously tracks many facial expression features, such as corners of the mouth, lips, and nose tip, but it is impossible to track the low texture area. Therefore, for a specific system, to effectively track the key low texture and obtain the coordinates of facial feature points and feature positions of each image, it is very important to form visual feature vectors.

## 4. Clustering and Learning

### 4.1. Clustering Algorithm

**Figure 6:**
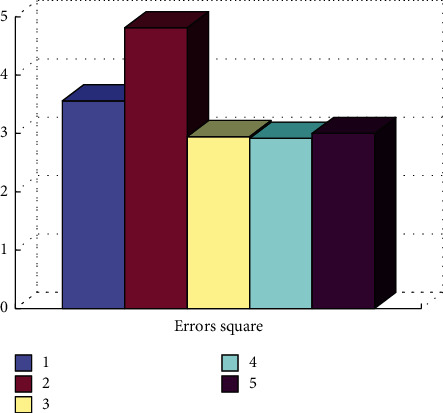
Comparison of clustering results.

Different clustering results can be obtained by adjusting the clustering algorithm parameters, such as the desired number of clusters, maximum training times, minimum number of samples per class, separation parameter *P*, and merging parameter *C*. These specific parameter values can be changed and calculated by formula (4). The specific results are shown in Figure 6.

(4) $$\begin{array}{c} \text{Error Square}\left(X,Y\right)=\frac{\sqrt{\left(X-Y\right)*{\left(X-Y\right)}^{T}}}{\left\|X\right\|}, \end{array}$$

where the real data matrix is represented by *X*, the matrix mapped from real data to category is represented by *Y*, and the matrix size is represented by ‖*X*‖.

### 4.2. Artificial Neural Network

**Figure 7:**
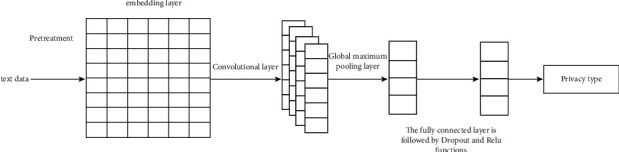
Structure diagram of neural network.

For artificial neural network, it is mainly learning-oriented input and output, which is used to reflect strong computational efficiency and corresponding robustness. The specific structure diagram is shown in Figure 7.

For each frame of speech, the 16-dimensional LPC and RASTA-PLP mixed vector and the 2-dimensional prosody parameters are calculated to form an 18-dimensional speech feature vector, and the front and back 8 frames are combined into an input vector. In this way, the input of each neural network is a vector.

## 5. Simulation Experiment

In order to cover the pronunciation of a single person as much as possible, this paper selects the sentence synchronous audio and video library in the Chinese speech synthesis library. By marking feature points, the motion data of lips, cheeks, and eyelids can be obtained. The camera converts the collected video into an image at 10 frames/*S* and uses the tracking program to process the image feature sequence.

**Figure 8:**
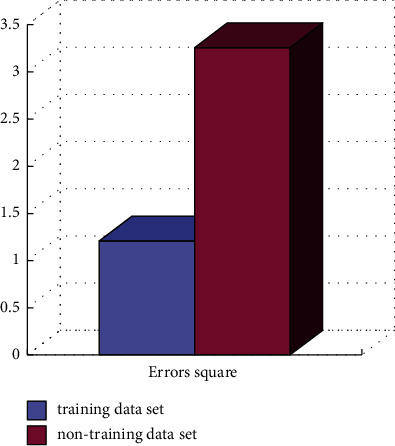
Variance comparison.

Both qualitative and quantitative evaluation methods are used for the system. Quantitative testing is based on calculation to measure the error between predicted data and real data. Most machine learning systems should use quantitative methods. Qualitative testing is to judge whether the synthesized face motion is true through perception. For synthesis, qualitative testing is very important. In quantitative testing, the specific calculated variance is shown in Figure 8.

The simulation results show that the compromised node detection in wireless sensor networks can not only solve the dynamic change of the upper part of the face but also use the recorded original voice and can effectively solve the synchronization problem. Therefore, it has been highly evaluated and proved to be effective in practice.

## 6. Conclusions

The continuous development of artificial intelligence promotes the improvement and enrichment of film and television animation. People are increasingly pursuing the aesthetic and technical characteristics of film and television animation. Therefore, more technologies are introduced into the field of film and television animation. In view of these needs and limitations, based on the compromised node detection of wireless sensor networks, this paper combs the synchronous business relationship flow of voice signals and facial expressions, analyzes the process of voice processing signals, first detects abnormal behavior and voice using compromise, finds the basic pattern of facial expressions through cluster analysis, and seems to complete the one-to-one mapping from voice to facial expressions. At the same time, quantitative and qualitative evaluation methods are used for analysis and evaluation. The prosodic distribution of speech features is used to realize the one-to-one mapping to facial expression, which avoids the defect of the robustness of speech recognition, improves the learning ability of speech recognition, and realizes the driving analysis of human facial expression film and television animation. The simulation results show that the compromised node detection in wireless sensor networks is effective and can support the analysis and research of speech-driven facial expression film and television animation.

## Data Availability

The labeled dataset used to support the findings of this study is available from the corresponding author upon request.
